# Partially Reduced Titanium Niobium Oxide: A High‐Performance Lithium‐Storage Material in a Broad Temperature Range

**DOI:** 10.1002/advs.202105119

**Published:** 2021-12-19

**Authors:** Tian Jiang, Siyuan Ma, Jianbin Deng, Tao Yuan, Chunfu Lin, Meilin Liu

**Affiliations:** ^1^ Institute of Materials for Energy and Environment School of Materials Science and Engineering Qingdao University Qingdao 266071 China; ^2^ School of Materials Science and Engineering University of Shanghai for Science and Technology Shanghai 200093 China; ^3^ School of Materials Science and Engineering Georgia Institute of Technology Atlanta GA 30332‐0245 USA

**Keywords:** anodes, in situ characterization, lithium batteries, low‐temperature operation, titanium niobium oxide

## Abstract

The existing electrode materials for lithium‐ion batteries (LIBs) generally suffer from poor rate capability at low temperatures, severely limiting their applications in winter and cold climate area. Here, partially reduced TiNb_24_O_62_ (PR‐TNO) are reported that demonstrates excellent electrochemical performance in a broad temperature range, notably at low temperatures. Its crystal structure is similar to that of Ti_2_Nb_10_O_29_ upon partial reduction in H_2_. The titanium and niobium ions in PR‐TNO enable multielectron transfer, safe operation, and high Coulombic efficiencies. Benefiting from the increased electronic conductivity of the partially reduced phase and its robust crystal structure with a large interlayer spacing, PR‐TNO shows fast electron and Li^+^ transport, small volume change associated with Li^+^ storage, and notable capacitive behavior, resulting in good electrochemical performance even at very low temperatures. At −20 °C, a large reversible capacity of 313 mAh g^−1^ is obtained at 0.1C, reaching 83.3% of that at 25 °C. At 5C, high rate capability (58.3% of that at 0.5C) is achieved, only slightly lower than that at 25 °C (60.7%). Furthermore, PR‐TNO demonstrates excellent cyclic stability with 99.2% of the initial capacity after 1680 cycles, confirming its excellent suitability for low‐temperature LIBs.

## Graphical abstract

A partially reduced TiNb_24_O_62_ electrode material demonstrates excellent electrochemical performance in a broad temperature range, notably at low temperatures. At −20 °C, it retains 83.3% of the reversible capacity at 25 °C, attributed to the fast electron‐ and Li^+^‐transport properties inherent to the Ti_2_Nb_10_O_29_‐type shear ReO_3_ crystal structure with lower‐valence cations and a large interlayer spacing.

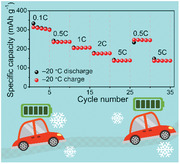

## 1 Introduction

Lithium‐ion batteries (LIBs) are widely used as power sources for a variety of devices, such as portable electronics, electric vehicles, and military equipment.^[^ ^1^ ^]^ However, many technical challenges still remain to meet the demand of a wide range of applications. In particular, poor performance of LIBs at low ambient temperature severely limits their applications in high altitudes, high latitudes, and deep space, because the rates of mass and charge transfer in LIBs decrease exponentially as the operation temperature is lowered.^[^ ^2^ ^]^ To overcome this problem, extra measures (e.g., external or internal heating of LIBs) have been explored to increase the operation temperature.^[^ ^3^ ^]^ However, these solutions are usually accompanied by increased system complexity, decreased energy efficiencies, and reduced energy/power density. Thus, it is highly desirable to develop an electrode material with good electrochemical performance at low temperatures.

Since the rates of mass and charge transfer decrease exponentially as the operation temperature is lowered, nanostructured and composite electrode materials have been created to improve the rate capabilities of LIBs.^[^ ^4^ ^]^ For instance, Yang et al. found that the obtainable capacity of 50–100 nm sized LiMn_0.8_Fe_0.2_PO_4_ particles (97 mAh g^−1^) was obviously larger than that of 100–150 nm sized particles (72 mAh g^−1^) at −15 °C due to the shorter solid‐state Li^+^‐transport distances in the smaller particles.^[^ ^4b^ ^]^ Similarly, TiO_2_ nanowires with ≈9 nm diameters synthesized by Li et al. maintained a capacity of ≈100 mAh g^−1^ at −25 °C, which was 50.2% of that at 25 °C, while nanowires with ≈100 nm diameters suffered from severe capacity decay and could only be used at small current rates.^[^ ^4c^ ^]^ Qin et al. combined Li_3_V_2_(PO_4_)_3_ nanoparticles with 4.67 wt% conductive graphite. Even at −30 °C, a discharge capacity of 90 mAh g^−1^ at 0.2C was achieved, corresponding to ≈80% of that at 25 °C.^[^ ^4a^ ^]^ 3.35 wt% carbon was coated on Li_4_Ti_5_O_12_ microparticles by Tao et al., and a discharge capacity of 108 mAh g^−1^ at 1C was obtained at −20 °C, which was only ≈10% smaller than that at 25 °C (119 mAh g^−1^).^[^ ^4d^ ^]^ However, the low tap density of nanomaterials and the complex fabrication processes of composite materials limit their practical applications. A more effective strategy is to significantly enhance the intrinsic transport properties of an electrode material in order to achieve excellent electrochemical performance at low temperatures.

In this work, we have successfully synthesized a partially reduced TiNb_24_O_62_ (PR‐TNO) fibers of submicron diameter through electrospinning and a subsequent calcination in H_2_ at 900 °C for 4 h. The crystal structure of the PR‐TNO sample is similar to that of Ti_2_Nb_10_O_29_. When used as an anode in an LIB, PR‐TNO demonstrates fast mass‐ and charge‐transport characteristics. First, the ReO_3_‐type layered crystal structure of PR‐TNO provides abundant room for fast Li^+^ transport and storage, and displays notable capacitive behavior especially at low temperatures.^[^ ^5^ ^]^ Second, PR‐TNO expands the interlayer spacing, which further facilitates the Li^+^ transport between layers.^[^ ^6^ ^]^ Third, the relatively high operation potential of PR‐TNO results in very thin solid electrolyte interphase (SEI) films coated on its primary particles,^[^ ^7^ ^]^ allowing fast Li^+^ transport between the electrolyte and the PR‐TNO lattice. Finally, the partially reduced titanium and niobium ions (such as Ti^3+^ and Nb^4+^) in PR‐TNO enhance the electronic conductivity.^[^ ^6a^ ^]^ The enhancements in these intrinsic properties enable the good electrochemical kinetics of PR‐TNO at low temperatures. At −20 °C, PR‐TNO delivers a large reversible capacity of 313 mAh g^−1^ at 0.1C, reaching 83.3% of that at 25 °C. PR‐TNO further shows high rate capability with a satisfactory 5C versus 0.5C capacity ratio of 58.3% at −20 °C, which is only slightly lower than that at 25 °C (5C vs 0.5C capacity ratio of 60.7%). Excitingly, PR‐TNO exhibits excellent cyclic stability with ultrahigh capacity retention of 99.2% after as long as 1680 cycles at 5C due to its very stable crystal structure with a maximum volume change of only 7.9%. In addition, the working mechanisms of PR‐TNO are systematically investigated through various state‐of‐the‐art characterizations, including variable temperature in situ X‐ray diffraction (XRD), in situ transmission electron microscopy (TEM), ex situ X‐ray photoelectron spectroscopy (XPS), ex situ high‐resolution transmission electron microscopy (HRTEM), and ex situ scanning transmission electron microscopy (STEM).

## 2 Results and Discussion

### 2.1 Physico‐Chemical Characterizations

**Figure 1:**
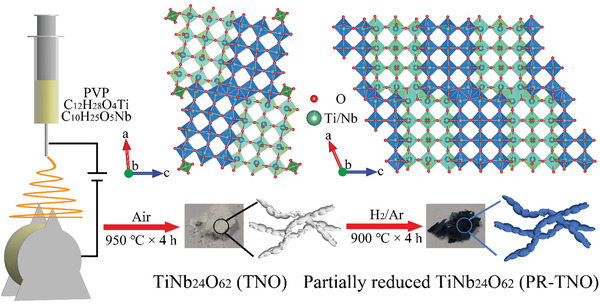
Preparation processes of TNO and PR‐TNO.

**Figure 2:**
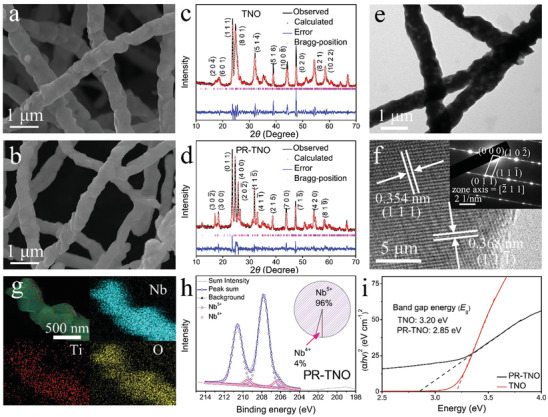
Physico‐chemical characterizations. FESEM images of a) TNO and b) PR‐TNO. XRD patterns of c) TNO and d) PR‐TNO with Rietveld refinements. e) TEM image, f) HRTEM with SAED pattern, g) EDX mapping images, and h) XPS spectrum of Nb element of PR‐TNO. i) Evolution of optical band gaps of TNO and PR‐TNO based on UV−vis absorption spectra.

The morphology of TNO prepared by an electrospinning method (**Figure** **1**) is submicron fibers with a diameter range of 350−700 nm (**Figure** **2**a), which is composed of tightly connected primary particles with sizes of ≈400 nm. After the H_2_ reduction (Figure 1), the morphology of PR‐TNO shows negligible change (Figure 2b,e), indicating that the fibers can withstand the high temperature of 900 °C in the reducing atmosphere. The Brunauer−Emmett−Teller (BET) specific surface area of PR‐TNO is determined to be a small value of 3.1 m^2^ g^−1^ (Figure S1, Supporting Information). The uniform fibers enable uniformly mixed electrode slurry, thus effectively avoiding the active‐material crushing on the working electrodes.

The XRD patterns of TNO and PR‐TNO are respectively shown in Figure 2c,d, and their Rietveld‐refined data are detailed in Tables S1 and S2 (Supporting Information).^[^ ^8^ ^]^ The XRD pattern of TNO matches with monoclinic TiNb_24_O_62_ (JCPDS No. 72–1655) having *C2* space group and no impurities.^[^ ^9^ ^]^ The high peak intensity indicates its high crystallinity. Its shear ReO_3_ crystal structure is shown in Figure 1. The structural unit is composed of a 3 × 4 octahedron‐block and 0.5 tetrahedron at the block corners with randomly distributed M (M = Ti and Nb ions with an atomic number ratio of 1: 24) in the centers of octahedra and tetrahedra, and the units extend infinitely toward the *b* axis. After 4 h of the H_2_ treatment, the O^2−^ loss turns TNO into PR‐TNO with a Ti_2_Nb_10_O_29_‐type structure (Figure 2d), and the color changes from white to navy, suggesting the generation of lower‐value cations (such as Ti^3+^ and Nb^4+^). As shown in Figure 1, the monoclinic shear ReO_3_ structure of PR‐TNO no longer has tetrahedra at the 3 × 4 octahedron‐block corners and the space group changes from *C2* to *A2/m* due to the decrease of the oxygen: metal ion ratio from 62/25 to 29/12.^[^ ^10^ ^]^ In the structures of TNO and PR‐TNO, the blue and green 3 × 4 MO_6_ octahedron‐block structural units are connected by the edge‐ and corner‐sharing of these octahedra at different layers, guaranteeing their good structural stability.^[^ ^11^ ^]^ In this type of layered structure, the lattice parameter *b* is equal to its interlayer spacing. It should be emphasized that the *b* value of PR‐TNO (0.3835 nm) is larger than that of TNO (0.3821 nm) and the previously reported M–Nb–O (M stands for metal ions) anode compounds with shear ReO_3_ structures (Table S3, Supporting Information), indicating the wider channels for fast Li^+^ transport and storage in PR‐TNO.

The lattice spacings in the HRTEM image of PR‐TNO (Figure 2f) are determined to be 0.368 and 0.354 nm, corresponding to the $\left(1\;1\;\bar{1}\right)$ and (1 1 1) crystallgraphic planes of PR‐TNO, respectively. The selected‐area electron diffraction (SAED) pattern with regular diffraction spots (Figure 2f, inset) reveals the single‐crystalline characteristic of the PR‐TNO primary particles, and can be assigned to the *A2/m* space group. Energy dispersive X‐ray (EDX) spectroscopy (Figure 2g) exhibits that Ti, Nb and O elements are homogeneously distributed in the tested fiber, conforming the homogeneous PR‐TNO crystals.

The full width‐scan XPS spectrum of PR‐TNO (Figure S2, Supporting Information) reveals the Nb, O and C (reference) elements, but the Ti element is almost undetectable probably due to its small mass ratio in PR‐TNO. The XPS high‐resolution spectrum of Nb is presented in Figure 2h. The binding energies centered at 210.6 and 207.8 eV match well with the Nb‐3*d*_5/2_ and Nb‐3*d*_3/2_ peaks of Nb^5+^, respectively,^[^ ^12^ ^]^ while those of 209.5 and 206.3 eV correspond to Nb^4+^.^[^ ^13^ ^]^ The Nb^4+^: Nb^5+^ peak‐area ratio is 4: 96, which is roughly consistent with the oxygen: metal: ion ratio in PR‐TNO (29/12). The 4% of Nb^4+^ together with the color change from white to navy clearly indicates that ≈4% of Nb^5+^ in TNO is reduced to Nb^4+^ after the H_2_ reduction. Meanwhile, the UV−vis absorption spectrum (Figure 2i) shows that the band gap is obviously decreased from 3.20 eV in TNO to 2.85 eV in PR‐TNO (Equation S1, Supporting Information), suggesting the much larger electronic conductivity of PR‐TNO. In fact, the tested electronic conductivity of PR‐TNO is 7.8 × 10^−5^ S cm^−1^, three orders of magnitude larger than that of TNO (2.6 × 10^−8^ S cm^−1^). In addition, the XPS high‐resolution spectrum of O (Figure S3, Supporting Information) reveals minor oxygen vacancies in PR‐TNO, suggesting that the phase transformation with the TiNb_24_O_62_‐type → Ti_2_Nb_10_O_29_‐type crystal‐structure change in this work largely consumes the oxygen vacancies created during the calcination of TiNb_24_O_62_ in the reducing atmosphere (H_2_), which is unlike the previous works showing that the calcination of TiNb_2_O_7_/Ti_2_Nb_10_O_29_ in inert atmospheres (Ar or N_2_) retained its crystal structure although plenty of oxygen vacancies were introduced.^[^ ^14^ ^]^

### 2.2 Li^+^‐Storage Performance

**Figure 3:**
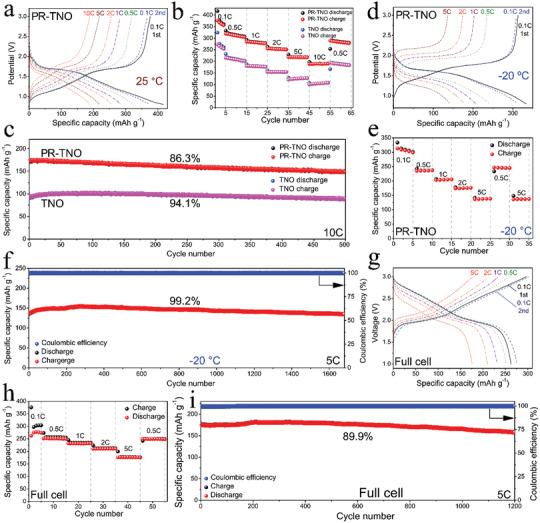
Li^+^‐storage performance. a) Discharge−charge curves of PR‐TNO/Li half cell at 25 °C. b) Rate capability and c) cyclic stability of PR‐TNO/Li and TNO/Li half cells at 25 °C. d) Discharge−charge curves, e) rate capability, and f) cyclic stability of PR‐TNO/Li half cell at −20 °C. g) Discharge−charge curves, h) rate capability, and i) cyclic stability of LiNi_0.8_Co_0.1_Mn_0.1_O_2_/PR‐TNO full cell at 25 °C.

**Figure** **3**a and Figure S4 (Supporting Information) respectively show the discharge−charge curves of PR‐TNO and TNO in the safe potential window of 3.0−0.8 V. The two sets of curves have similar shapes with pseudo‐plateaus at 1.7−1.6 V and operation potentials averaging at ≈1.45 V, which is the lowest among the known M−Nb−O anode materials^[^ ^5^, ^15^ ^]^ and smaller than that of Li_4_Ti_5_O_12_ (≈1.57 V).^[^ ^7^ ^]^ The pseudo‐plateau in each discharge/charge curve corresponds to a two‐phase reaction, while two sloping regions respectively within 3.0−1.7 and 1.6−0.8 V indicate two different solid‐solution reactions.^[^ ^5a^ ^]^ The first‐cycle Coulombic efficiency and reversible capacity of PR‐TNO are as large as 90.2% and 376 mAh g^−1^, obviously larger than those of TNO (84.3% and 273 mAh g^−1^). It is noteworthy that PR‐TNO owns the largest capacity among the M−Nb−O anode materials developed so far (Tables S3 and S4, Supporting Information). When the current rate is increased to 0.5C, 1C, 2C, 5C, and 10C, the reversible capacities of PR‐TNO retain 313, 282, 253, 218, and 190 mAh g^−1^ (Figure 3b), respectively, indicating its high rate capability, which is significantly higher than that of TNO with only 105 mAh g^−1^ at 10C. When the current rate returns from 10C to 0.5C, the capacity of PR‐TNO is slightly decreased. When continuing to test 500 cycles at 10C, large capacity retention of 86.4% (Figure 3c) is achieved. Clearly, the H_2_ reduction treatment of TNO significantly improves the reversible capacity and rate capability while retaining the safe operation potential and good cyclic stability.

The electrochemical performance of the PR‐TNO/Li half cell is further examined at the low temperature of −20 °C. The low‐temperature discharge/charge platform slightly drops/rises by ≈0.1 V and the average operation potential slightly increases by 0.1 V compared with those at 25 °C (Figure 3d). In addition, the high‐potential electrochemical reaction at the low temperature is less intensive, resulting in a thinner SEI‐film thickness on the PR‐TNO primary particles after 50 cycles at 10C (≈1.0 nm at −20 °C vs ≈1.3 nm at 25 °C, Figure S5, Supporting Information). During the first cycle at 0.1C, the reversible capacity retains 313 mAh g^−1^, which is up to 83.3% of that at 25 °C and even larger than those of most M−Nb−O at 25 °C (Tables S3 and S4, Supporting Information). The first‐cycle Coulombic efficiency increases by 3.8%, reaching an ultralarge percentage of 94.0%. The reversible capacities at 0.5C, 1C, 2C, and 5C respectively retain 236, 205, 175 and 138 mAh g^−1^ (Figure 3e), which are 75.5, 72.6, 69.2, and 63.1% of the corresponding values at 25 °C. The 5C versus 0.5C capacity ratio at −20 °C reaches 58.3%, which is slightly smaller than that at 25 °C (60.7%). Therefore, the low‐temperature rate capability of PR‐TNO is also satisfactory when considering −20 °C is a rather low temperature. Furthermore, after 1680 cycles at 5C, a very stable capacity of 137 mAh g^−1^ and ultrahigh capacity retention of 99.2% are obtained (Figure 3f). This better cyclic stability at −20 °C than that at 25 °C is attributed to the smaller utilization of the reversible capacity at the lower temperature. To sum up, the low‐temperature operation of PR‐TNO retains its safe operation potential, slightly decreases its reversible capacity and rate capability, but improves its first‐cycle Coulombic efficiency and cyclic stability.

To prove the practicability of PR‐TNO, the PR‐TNO anode is coupled with a LiNi_0.8_Co_0.1_Mn_0.1_O_2_ cathode, and the resultant LiNi_0.8_Co_0.1_Mn_0.1_O_2_/PR‐TNO full cell is examined. It yields a large discharge capacity of 277 mAh g^−1^ with an average operation voltage of 2.26 V at 0.1C (Figure 3g). At 0.5C, 1C, 2C, and 5C, the reversible capacity remains 251, 231, 211, and 177 mAh g^−1^, respectively (Figure 3h). After 1200 cycles at 5C, it still remains 158 mAh g^−1^ with high capacity retention of 89.9% (Figure 3i). The above electrochemical results of both half and full cells demonstrate that PR‐TNO is a practical anode material with a large reversible capacity, high first‐cycle Coulombic efficiency, safe operation potential, high rate capability and good cyclic stability at both room and low temperatures.

### 2.3 Redox Mechanism and Electrochemical Kinetics

**Figure 4:**
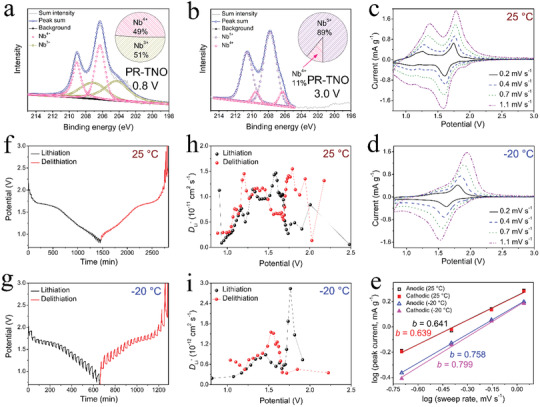
Redox mechanism and electrochemical kinetics. Ex situ XPS spectra of Nb element in PR‐TNO at a) lithiation to 0.8 V and b) delithiation to 3.0 V states. CV curves of PR‐TNO/Li half cells at c) 25 and d) −20 °C. e) Determination of *b*‐values using log (peak current) versus log (sweep rate) relationship for PR‐TNO/Li half cells at 25 and −20 °C. GITT curves of PR‐TNO/Li half cells at f) 25 and g) −20 °C. Variations in Li^+^ diffusion coefficient of PR‐TNO at h) 25 and i) −20 °C.

In order to investigate the reasons for the comprehensively good Li^+^‐storage performance of PR‐TNO at room and low temperatures, various in‐depth characterizations are carried out. The valence variations of Nb in PR‐TNO are revealed in its ex situ XPS spectra. After discharge to 0.8 V, the higher doublet at 209.5 and 206.3 eV can be assigned to Nb‐3d_3/2_ and Nb‐3d_5/2_ of Nb^4+^,^[^ ^13^ ^]^ and the lower doublet at 207.1 and 204.3 eV can correspond to Nb^3+^ (**Figure** **4**a),^[^ ^16^ ^]^ which indicate that the two‐electron transfer per Nb indeed occurs during the electrochemical reaction of PR‐TNO. The peak‐area ratio of the former and latter doublets is 49: 51, suggesting that 49% of Nb^4+^ and 51% of Nb^3+^ coexist, which match with the capacity of PR‐TNO. After charge to 3.0 V, the valence of Nb recovers to a great extent, achieving 89% of Nb^5+^ and 11% of Nb^4+^ (Figure 4b). Compared with the pristine PR‐TNO (Figure 2h), the Nb^4+^ content increases by only 7% after the first cycle. This slight increase is reasonable since the inserted Li^+^ ions cannot be fully extracted during the first charge process (the first‐cycle Coulombic efficiency of PR‐TNO is 90.2%).^[^ ^5a^ ^]^ Therefore, the good multi‐electron electrochemical reversibility can be identified in PR‐TNO.

The cyclic voltammetry (CV) experiments are performed on the PR‐TNO/Li half cell at various sweep rates within 0.8−3.0 V, revealing the redox mechanisms and capacitive contributions in PR‐TNO at 25 and −20 °C. At 0.2 mV s^−1^ and 25 °C (Figures S6a and S4c, Supporting Information), three CV‐peak pairs respectively located at ≈1.9/≈1.8, ≈1.7/≈1.6, and ≈1.3/≈1.2 V can be attributed to the redox reactions based on the Ti^3+^/Ti^4+^, Nb^4+^/Nb^5+^, and Nb^3+^/Nb^4+^ couples, respectively.^[^ ^9^, ^17^ ^]^ At −20 °C (Figures S6b and S4d, Supporting Information), the CV peaks at ≈1.3/≈1.2 V become weak as a result of the smaller capacity at the low temperature. With the increase of the sweep rates (*v*), the value of peak current (*I*) keeps increasing, which can conform to the equation of *I* = *aν* ^*b*^.^[^ ^18^ ^]^ In this equation, *a* and *b* are adjustable parameters. The *b* value should be in a range of 0.5−1, and a larger *b*‐value suggests a larger proportion of capacitive contribution. Since the capacitive behavior is not controlled by solid‐state diffusion, it enables fast charge transport.^[^ ^18^ ^]^ The calculated *b* values of the anodic/cathodic peak at 25 and −20 °C are 0.641/0.639 and 0.758/0.799, respectively (Figure 4e), indicating that the capacitive contribution in the Li^+^‐storage process of PR‐TNO is rather limited at 25 °C but becomes significant at −20 °C, which benefits its low‐temperature electrochemical kinetics.

To investigate the Li^+^‐transport kinetics of PR‐TNO, its Li^+^ apparent diffusion coefficients (*D*_Li_) at various states of discharge/charge are calculated based on the galvanostatic intermittent titration technique (GITT) data recorded at 25 and −20 °C (Figure 4f,g, Equation S2, Supporting Information).^[^ ^19^ ^]^ During lithiation, the average *D*_Li_ value of PR‐TNO is 7.4 × 10^−12^ cm^2^ s^−1^, and the corresponding value during delithiation is 8.9 × 10^−12^ cm^2^ s^−1^ (Figure 4h), which are among the best results in the M–Nb–O field (Table S5, Supporting Information). When the temperature drops to −20 °C (Figure 4i), the *D*_Li_ values decrease by only one order of magnitude, retaining 8.3 × 10^−13^ cm^2^ s^−1^ (lithiation) and 8.2 × 10^−13^ cm^2^ s^−1^ (delithiation). Surprisingly, the overall apparent Li^+^ diffusion coefficient of PR‐TNO at such low temperature of −20 °C is even larger than most of the previously developed M−Nb−O anode materials at room temperature (Table S5, Supporting Information). This fast Li^+^ transport in PR‐TNO is undoubtedly ascribed to the criss‐cross Li^+^ transport network in the 3 × 4 × ∞ shear ReO_3_‐type layered structure with the large interlayer spacing. It should be emphasized that the specific surface area of PR‐TNO is rather small, suggesting that the material nanosizing effects on its electrochemical performance are very limited. Based on the above kinetics characterizations and analyses, it can be concluded that the intrinsically fast Li^+^/electron transport and the notable capacitive behavior in PR‐TNO work together to achieve its advanced low‐temperature rate capability.

### 2.4 Li^+^‐Storage Mechanisms

**Figure 5:**
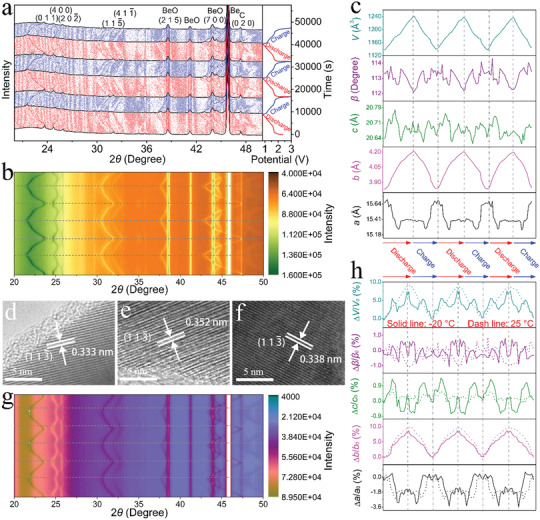
Crystal‐structural evolution. a) Pristine in situ XRD patterns of PR‐TNO/Li in situ cell with corresponding discharge−charge curves within 3.0–0.8 V at 0.3C and 25 °C (initial three cycles). b) 2D in situ XRD patterns of PR‐TNO/Li in situ cell at 25 °C. c) Lattice‐parameter variations of PR‐TNO at 25 °C (initial three cycles). Ex situ HRTEM images of PR‐TNO at d) pristine, e) lithiation to 0.8 V, and f) delithiation to 3.0 V states. g) 2D in situ XRD patterns of PR‐TNO/Li in situ cell at −20 °C. h) Comparisons of lattice‐parameter variations (in percentage) of PR‐TNO at −20 °C (solid line) and 25 °C (dash line).

In order to explore the crystal‐structural evolution of PR‐TNO during the electrochemical reaction, an in situ XRD characterization of a PR‐TNO/Li in situ cell is performed during the first three lithiation−delithiation cycles at 0.3C and 25 °C (**Figure** **5**a,b). When lithiation from the open circuit voltage to ≈1.7 V (i.e., the first solid‐solution reaction), all the XRD peaks of the original phase shift to smaller Bragg angles, and their peak intensities gradually decrease since the Li^+^ insertion into the PR‐TNO lattice undoubtedly decreases its crystal‐structural order. In the following pseudo‐plateau within 1.7–1.6 V, the (0 1 1), (2 0 $\bar{2}$), (1 1 $\bar{5}$), (4 1 $\bar{1}$), (2 1 5), and (0 2 0) peaks continue shifting to smaller angles, while the (4 0 0) and (7 0 0) peaks shift to larger angles. Interestingly, these peaks significantly broaden and weaken, confirming the two‐phase reaction at this stage. When continuing lithiation from ≈1.6 to 0.8 V (i.e., the second solid‐solution reaction), all the XRD peaks of the new phase shift to smaller angles and gradually weaken. The evolution of all these XRD peaks is highly reversible in the subsequent delithiation process, and they almost fully recover their original positions and intensities at 3.0 V. In the second and third cycles, the peak evolution is almost the same as that in the first cycle, demonstrating the intercalation‐type nature of PR‐TNO with excellent structural and electrochemical reversibility during repeated lithiation and delithiation.

The lattice parameters *a*, *b*, *c*, *β* and *V* of PR‐TNO at various states of discharge/charge are determined by the Rietveld refinements of the in situ XRD patterns (Figure 5c), exhibiting high reversibility during lithiation–delithiation. During lithiation, the *a*‐value variation roughly follows a sequence of tiny increase → small decrease → tiny increase → small decrease → small increase → stable. The maximum *a*‐value change is −2.5% at ≈1.5 V, and the change becomes −1.4% at 0.8 V. Meanwhile, the *c* and *β* values show complex and subtle changes with only +0.1 and −0.9% at 0.8 V, respectively. In contrast, the *b* value monotonically and obviously increases, reaching a maximum change of +9.7%. Consequently, the maximum *V*‐value change is calculated to be +9.1%. These variations in the lattice parameters are verified by the ex situ HRTEM characterization of PR‐TNO, which reveals that the (1 1 $\bar{3}$) lattice spacing increases from 0.333 nm (pristine state, Figure 5d) to 0.352 nm (lithiation state at 0.8 V, Figure 5e) and turns back to 0.338 nm (delithiation state at 3.0 V, Figure 5f). It is noteworthy that the maximum unit‐cell‐volume change of PR‐TNO is much smaller than those of TNO (+17.5%) and graphite (+13.2%),^[^ ^20^ ^]^ attributed to their different layered crystal structures with different interlayer spacings.

The in situ XRD characterization of the PR‐TNO/Li in situ cell at −20 °C also reveals good reversibility of the peak evolution (Figures S8 and S5g, Supporting Information) and lattice‐parameter variations (Figure 5h). The *c* and *β* values at −20 °C show slightly larger variations compared with those at 25 °C. The maximum *b*‐value change decreases from +9.7% at 25 °C to +8.7% at −20 °C, but unexpectedly that for the *a* value increases from −2.5% at 25 °C to −3.1% at −20 °C. Consequently, the *V*‐value variation during lithiation at −20 °C follows a sequence of increase → small decrease → increase → tiny decrease with a maximum change of +7.9%, which is obviously smaller than that at 25 °C (+9.1%). This decrease of the maximum volume change is reasonable since the reversible Li^+^‐storage capacity at the low temperature is smaller (Figure 3d), and undoubtedly results in the better cyclic stability at the low temperature (Figure 3f).

**Figure 6:**
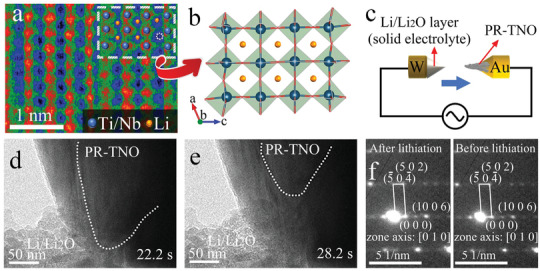
STEM and in situ TEM characterizations. Spherical‐aberration‐corrected STEM results of lithiated PR‐TNO (0.8 V) confirming its Li^+^‐storage sites. a) Magnified ABF STEM color image with inverted image contrast showing the positions of inserted Li^+^ ions (an example is indicated by a dotted circle). b) Crystal structure of lithiated PR‐TNO viewing along [0 1 0] zone axis. c) Schematic illustration of in situ TEM battery setup. In situ TEM characterization of PR‐TNO during lithiation: time‐lapse TEM images of Li^+^ insertion into PR‐TNO at d) 22.2 and e) 28.2 s. White dotted lines indicate reaction fronts inside PR‐TNO. f) SAED patterns of PR‐TNO at pristine and lithiation states.

Although the variation trends of the lattice parameters at 25 and −20 °C are not the same, the *V*‐value variation is mainly controlled by the *b*‐value variation in both cases. Therefore, it can be deduced that the external Li^+^ ions are mainly located within the interlayers between the *ac*‐layers, which is verified by the following spherical‐aberration‐corrected STEM characterization. The high‐angle annular dark field (HAADF) STEM image of lithiated PR‐TNO (Figure S7a, Supporting Information) viewed along the [0 1 0] zone axis reveals the excellent structural stability of PR‐TNO. Based on the annular bright field (ABF) STEM image (Figure S7b, Supporting Information), the exact Li^+^‐storage sites can be determined, which has been regarded as a powerful technique to directly observe Li^+^ within electrode materials.^[^ ^21^ ^]^ In order to make Li^+^ clear, the ABF STEM image is magnified, and the image contrast is inverted (**Figure** **6**a). The Li^+^‐storage sites are thus visible, and an example is highlighted by the dotted circle, demonstrating that the Li^+^ ions indeed occupy the interstices between the (0 1 0) planes of the PR‐TNO lattice, as schematically illustrated in Figure 6b.

In situ TEM is used to further investigate the volume variation and structural evolution of PR‐TNO during the electrochemical reaction. With increasing of in situ biasing (Figure 6c), the Li^+^ ions extract from the Li metal, then pass through the Li_2_O solid electrolyte, and finally insert into the PR‐TNO lattice. The lithiation reaction front sweeps across the PR‐TNO primary particle without obvious volume variations or fractures (Figure 6d,e, Supporting Information Video), confirming the intercalation nature of PR‐TNO with small volume change during lithiation. In addition, the comparison of the SAED patterns before and after lithiation (Figure 6f) shows no obvious changes, verifying the small variations in the lattice parameters *a* and *c*. All these in situ TEM results coincide with the aforementioned in situ XRD and ex situ STEM characterizations.

## 3 Conclusion

It is demonstrated that partially reduced TiNb_24_O_62_ (PR‐TNO) is a promising anode material for low‐temperature operation. Upon calcination in a reducing atmosphere, the crystal structure of PR‐TNO is changed to a Ti_2_Nb_10_O_29_‐type shear ReO_3_ structure with a large interlayer spacing of 0.3835 nm, allowing fast Li^+^ transport (8.3 × 10^−13^ cm^2^ s^−1^ during lithiation and 8.2 × 10^−13^ cm^2^ s^−1^ during delithiation) and significant capacitive behavior at −20 °C. The excellent electrochemical performance is attributed mainly to the enhancements in intrinsic properties of PR‐TNO phase, including increased ionic and electronic conductivity as well as improved capacity. The fast Li^+^ and electron transport enables high rate capability, achieving a satisfactory 5C versus 0.5C capacity ratio of 58.3% at −20 °C, which approaches that at 25 °C (60.7%). The highly reversible redox reactions of the Nb^4+^/Nb^5+^ and Nb^3+^/Nb^4+^ couples are confirmed in PR‐TNO. Consequently, at −20 °C, a large reversible capacity (313 mAh g^−1^ at 0.1C, 83.3% of that at 25 °C), safe operation potential (≈1.55 V), high first‐cycle Coulombic efficiency (94.0%), and very thin SEI films (≈1.0 nm thickness after 50 cycles at 10C) are achieved. When the majority of Li^+^ ions insert into/extract from the interstices between the (0 1 0) planes, the maximum unit‐cell‐volume expansion/shrinkage is only 7.9%, resulting in excellent cyclic stability (ultrahigh capacity retention of 99.2% after 1680 cycles at 5C) at −20 °C. The methodology and insight gained in this study may be applicable to structure and composition design of other high‐performance electrodes for low‐temperature operation.

## 4 Experimental Section

### Material Preparations

The preparation processes of TiNb_24_O_62_ (TNO) and partially reduced TiNb_24_O_62_ (PR‐TNO) are illustrated in Figure 1. The TNO submicron fibers were prepared by an electrospinning method. 0.19 mmol titanium isopropoxide (C_12_H_28_O_4_Ti, Aladdin, 99.99%) was dissolved in 15 mL ethanol. Then, 4.5 mmol niobium ethoxide (C_10_H_25_O_5_Nb, Aladdin, 98%) was dissolved into the above ethanol solution (total solute mass was 1.5 g). After stirring for 2 h, 0.6 g of polyvinylpyrrolidone (PVP, Macklin, *M*_w_ = 1 300 000) was slowly dissolved to the solution with continuous stirring for 5 h to obtain a clear, transparent, and viscous solution with a light yellow color. All the configurations of the above precursor solution were carried out in an Ar atmosphere. Then, the precursor solution was loaded into a 10 mL syringe and pumped through an 18 G needle at 2.1 mL h^−1^. The provided voltage was 10 kV, and the spun fibers were collected in a dynamic collector (Al foil) located 10 cm below the spinneret. The collected fibers were sintered at 950 °C for 4 h. The obtained TNO fibers with a white color were sintered at 900 °C for 4 h in a H_2_/Ar (1/9) atmosphere, and a sample of PR‐TNO submicron fibers with a navy color was finally achieved. To prepare the samples for electronic‐conductivity tests, Nb_2_O_5_ (Aladdin, 99.9%) and TiO_2_ (Aladdin, 99.99%) powders after ball milling in a SPEX‐8000M miller for 1 h were first pelletized under 20 MPa for 90 s and calcined in air at 1000 °C for 6 h, forming TNO pellets with thicknesses of ≈1.5 mm. The TNO pellets were then calcined at 950 °C in H_2_/Ar for 15 h, forming RP‐TNO pellets. Platinum was finally evaporated onto the upper and lower surfaces of the pellets, achieving Pt/TNO/Pt and Pt/PR‐TNO/Pt ion‐blocking cells (Figure S9, Supporting Information).

### Material Characterizations

The crystal structures of the materials were characterized by X‐ray diffraction (XRD). The powder XRD patterns were recorded on an Ultra IV diffractometer with Cu‐K*α* radiation (*λ* = 0.15418 nm) over a 2*θ* range of 10−70°. The Rietveld‐refinements of the XRD patterns were performed on the free GSAS program.^[^ ^8^ ^]^ The compositions and chemical valences of the PR‐TNO samples were measured using X‐ray photoelectron spectroscopy (XPS) on a Thermo Fisher Escalab 250XI spectrometer. Before the XPS measurements, the PR‐TNO particle surfaces were etched using focused argon‐ion sputtering. The absorbance of the materials in a wavelength range of 200−800 nm was measured using a SolidSpec 3600 Plus UV−Vis spectrophotometer. Field emission scanning electron microscopy (FESEM) analyses at 10 kV were conducted on a JEOL JSM‐7800F microscope to study the morphologies of the samples. Transmission electron microscopy (TEM) analyses were performed on a JEOL JEM‐2100 microscope to further study the crystal structures and morphologies. The BET specific surface area of PR‐TNO was obtained on an ASAP 2460 surface area analyzer. The HAADF and ABF STEM images were recorded on a Titan Cubed Themis G2 300 spherical‐aberration‐corrected microscope. The electronic conductivities of TNO and PR‐TNO were measured by a two‐probe direct current method. Their measurements were performed on their ion‐blocking cells by using a Chenhua CHI660E electrochemical workstation under 100 mV.

### Electrochemical Tests

The electrochemical performance of TNO and PR‐TNO at room temperature (25 °C) and low temperature (−20 °C) was examined using CR 2016‐type coin cells, which were assembled in an argon‐filled glove box. In each half cell, a Celgard 2325 microporous polypropylene film and lithium foil were respectively employed as the separator and counter electrode, and a mixture of diethylene carbonate, ethylene carbonate, and dimethyl carbonate (1: 1: 1 in volume) with 1 M LiPF_6_ served as the electrolyte. The working electrode consisted of a dried‐mixture film on a Cu foil. The mixture was obtained by homogeneously mixing the active material (TNO or PR‐TNO, 70 wt%), binder (polyvinylidene fluoride, 10 wt%) and carbon (Super P, 20 wt%). For the full cell, LiNi_0.8_Co_0.1_Mn_0.1_O_2_ (Shenzhen BTR New Energy Material Co. Ltd., M8‐S) and PR‐TNO were respectively the cathode and anode materials with loading densities of 2.5 and 1.0 mg cm^−2^. The preparation process of the LiNi_0.8_Co_0.1_Mn_0.1_O_2_ cathode was similar to that of the PR‐TNO anode, but an Al foil was used as the current collector. The galvanostatic discharge–charge (GCD) and galvanostatic intermittent titration technique (GITT) tests were performed on a Neware CT‐3008 eight‐channel battery testing system. Here, the current rate of 1C for both TNO and PR‐TNO was equivalent to a current density of 402 mA g^−1^, corresponding to the theoretical capacity of TiNb_24_O_62_ (402 mAh g^−1^). The cyclic voltammetry (CV) tests were conducted on the above electrochemical workstation. The low‐temperature tests were completed in a LINPIN LRHS‐225B‐LJ atmosphere chamber.

### In Situ Characterizations

In situ half cells for in situ XRD characterizations (LIB‐LHTXRD‐LN, Beijing Scistar Technology Co., Ltd.) equipped with a Be window and a fiberglass separator per cell were assembled to study the crystal‐structural evolution of PR‐TNO. The real‐time observation of morphological and structural evolution of PR‐TNO was achieved by in situ TEM on an electrochemical holder developed by X‐mech Center (Zhejiang University).^[^ ^22^ ^]^ A PR‐TNO fiber and Li metal were respectively located on the front of gold and tungsten rods to be used as the two electrodes of the in situ TEM half cell. The formed Li_2_O coating on Li metal acted as solid electrolyte. When the two electrodes touched each other, lithiation reaction was driven to occur by an applied negative voltage of −3 V.

## Conflict of Interest

The authors declare no conflict of interest.

## Supporting information

Supporting Information

Supplemental Video 1

## Data Availability

Research data are not shared.
