# Supplementary material for: Partially Reduced Titanium Niobium Oxide: A High‐Performance Lithium‐Storage Material in a Broad Temperature Range
Source: Adv Sci (Weinh). 2021 Dec 19;9(5):2105119. doi: 10.1002/advs.202105119 (PMC8844579; doi:10.1002/advs.202105119)
Supplement: Supplementary file 1 — Supporting Information [file ADVS-9-2105119-s001.pdf]

WILEY-VCH

ADVANCED SCIENCE
Open Access

Supporting Information

for *Adv. Sci.*, DOI: 10.1002/advs.202105119

Partially Reduced Titanium Niobium Oxide: A High-Performance Lithium-Storage Material in a Broad Temperature Range

Tian Jiang, Siyuan Ma, Jianbin Deng, Tao Yuan, Chunfu Lin* and Meilin Liu**

Supporting Information

Partially Reduced Titanium Niobium Oxide: A High-Performance Lithium-Storage Material in a Broad Temperature Range

Tian Jiang, Siyuan Ma, Jianbin Deng, Tao Yuan, Chunfu Lin* and Meilin Liu**

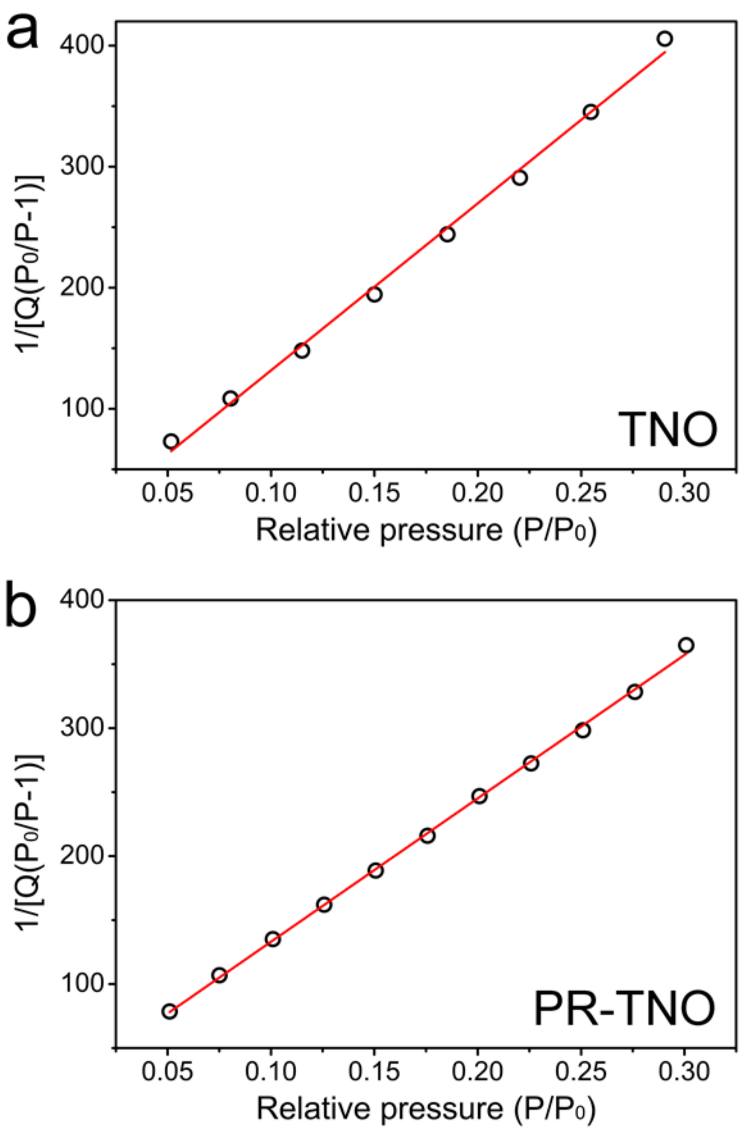

Figure S1. BET specific surface area plots of a) TNO and b) PR-TNO, revealing that the BET specific surface area of PR-TNO (3.1 m^2 g^{-1}) is very similar to that of TNO (3.2 m^2 g^{-1}).

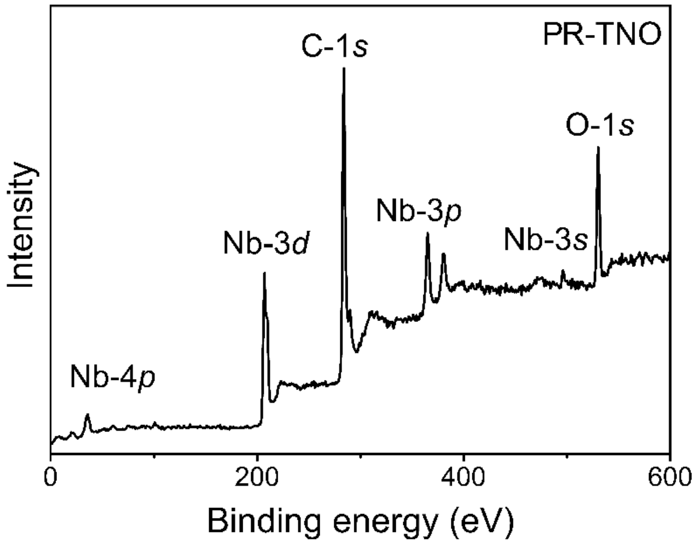

Figure S2. XPS survey spectrum of PR-TNO.

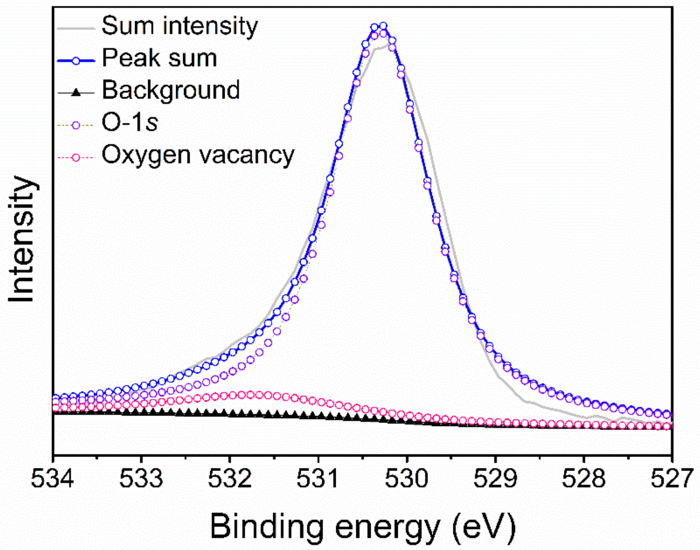

Figure S3. XPS spectrum of O element of PR-TNO. Characteristic peaks at 530.3 and 531.7 eV correspond to lattice oxygen and oxygen vacancies, respectively. The very weak peak at 531.7 eV indicates minor oxygen vacancies in PR-TNO.

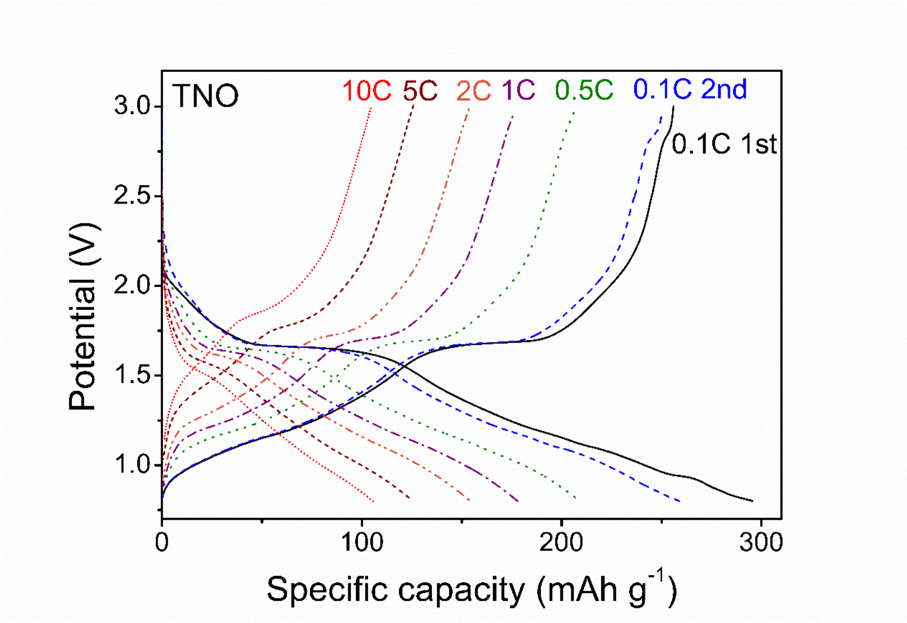

Figure S4. Discharge–charge curves of TNO at various current rates.

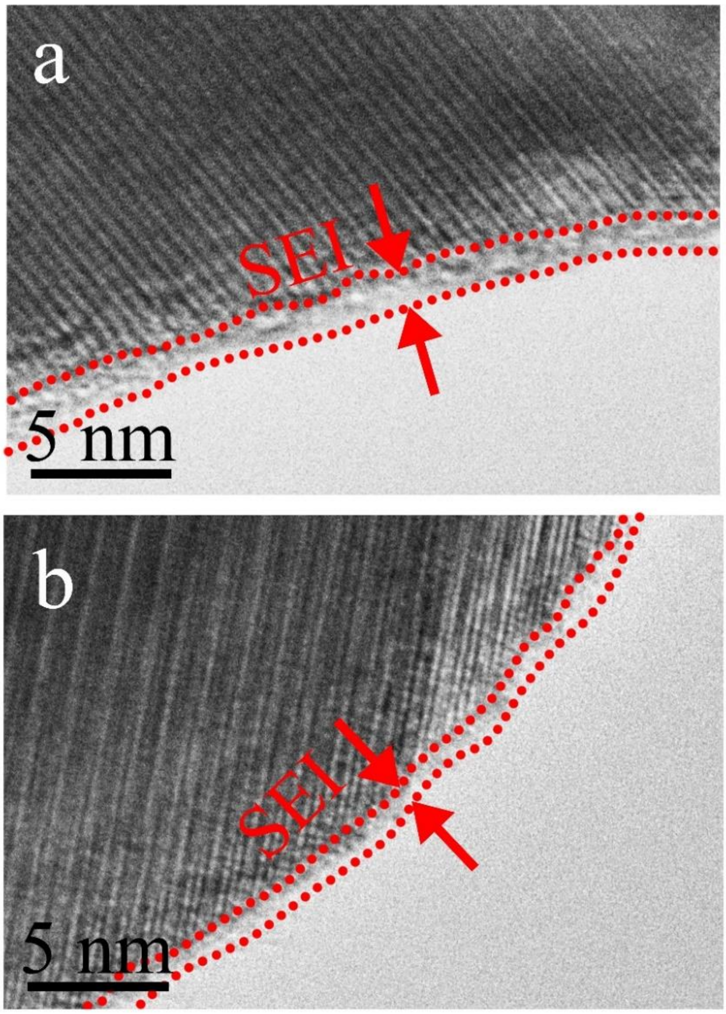

Figure S5. HRTEM images of PR-TNO showing SEI films after first 50 cycles at a) 25 and b) −20 °C.

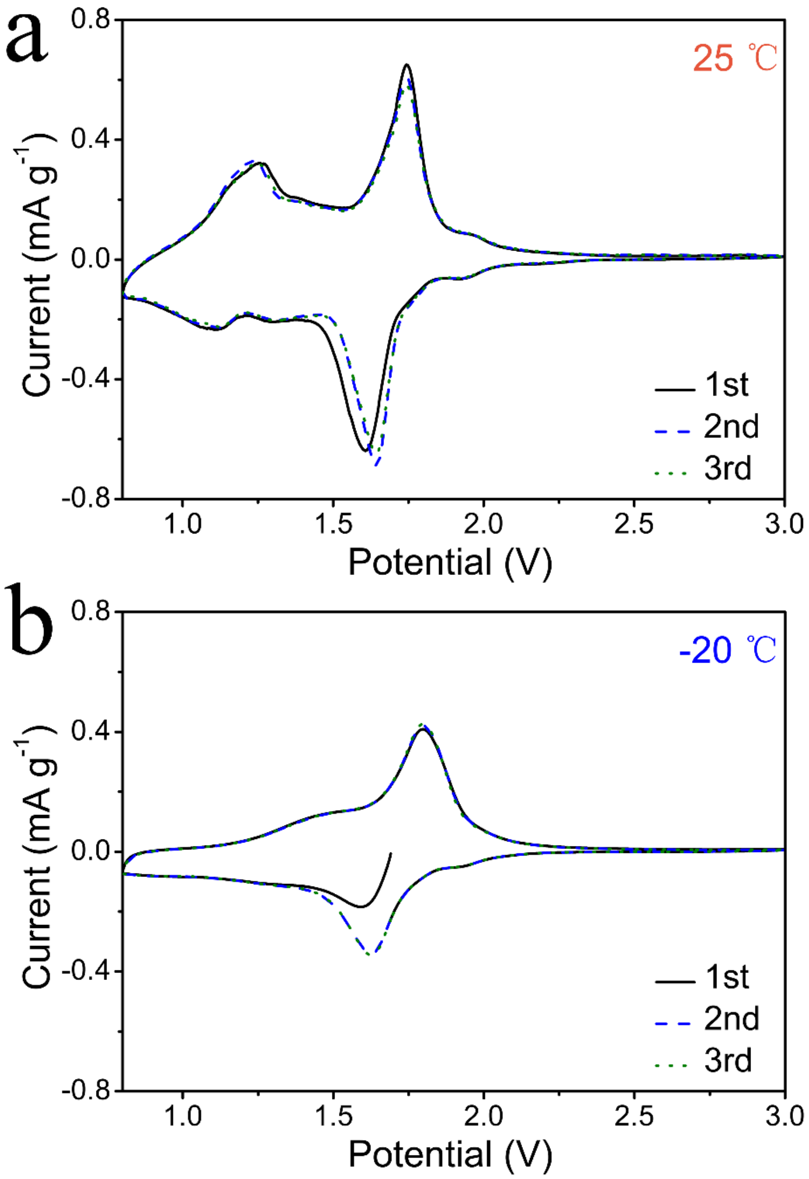

Figure S6. First three CV cycles at 0.2 mV s^{-1} of PR-TNO/Li half cell at a) 25 and b) −20 °C.

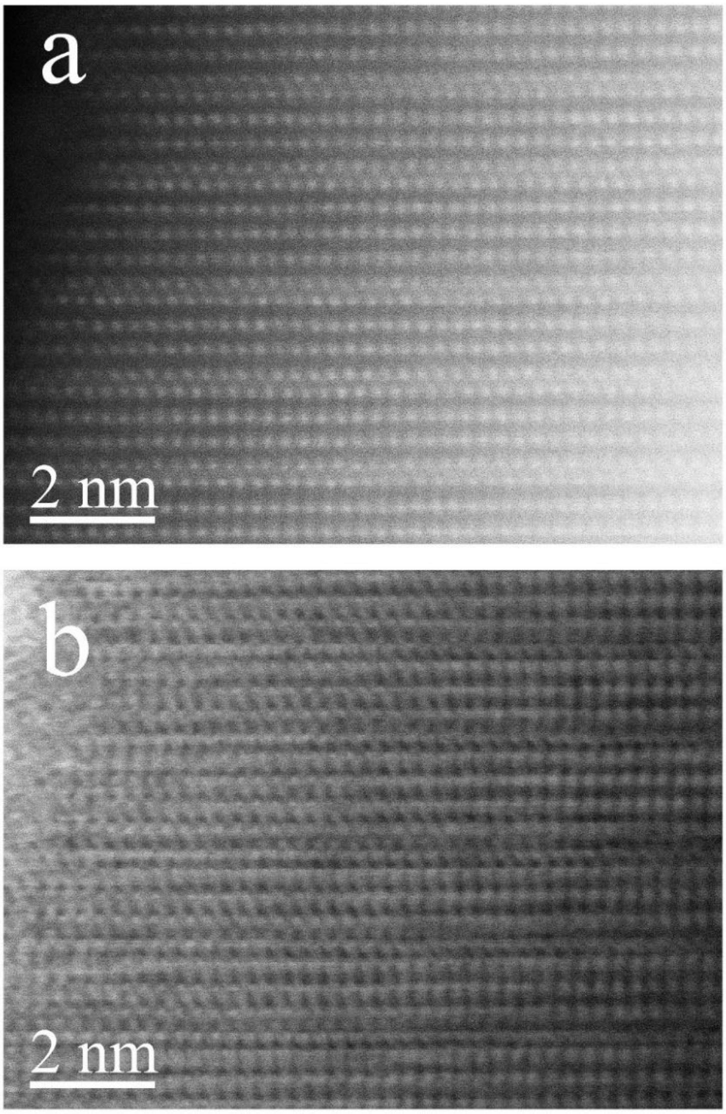

Figure S7. a) HAADF STEM and b) ABF STEM images of lithiated PR-TNO (0.8 V) viewed along [0 1 0].

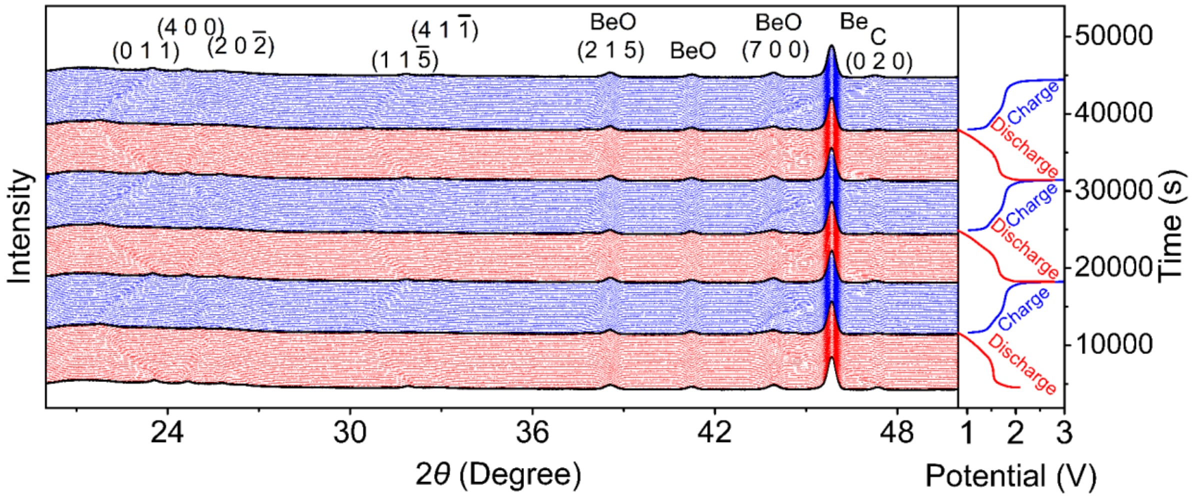

Figure S8. Pristine *in-situ* XRD patterns of PR-TNO/Li *in-situ* cell with corresponding discharge−charge curves within 3.0–0.8 V at 0.3C and –20 °C (initial three cycles).

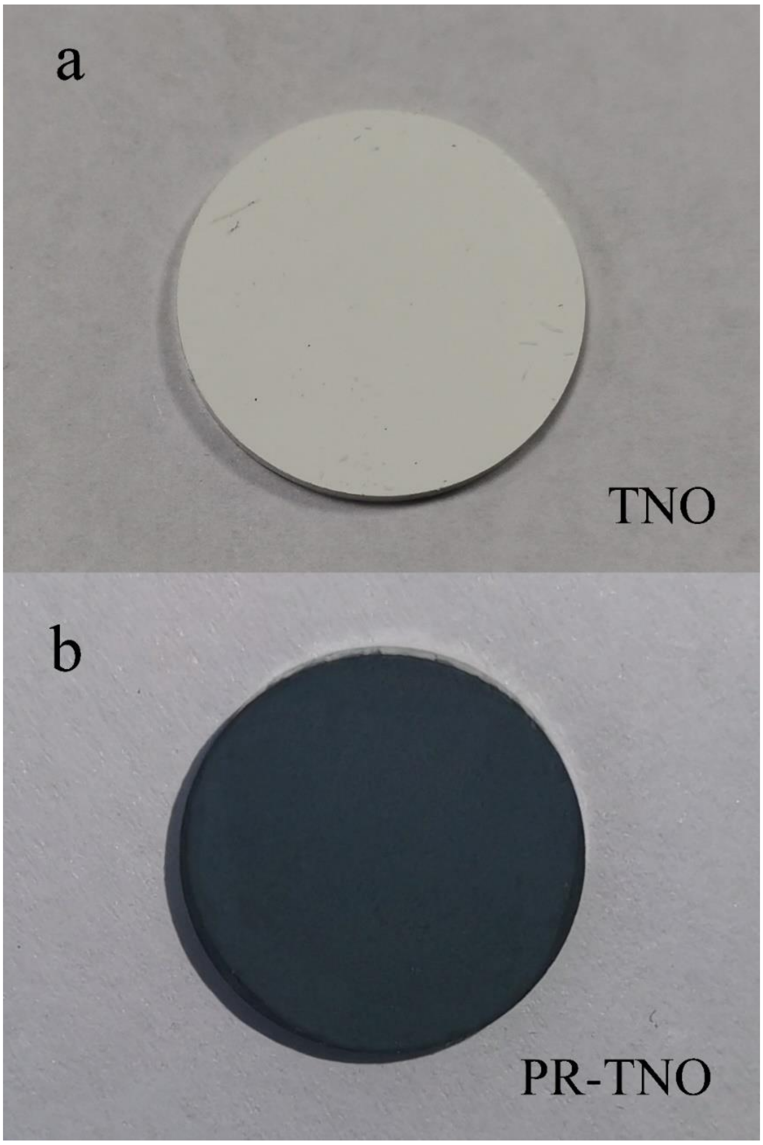

Figure S9. Digital photos of a) Pt/TNO/Pt and b) Pt/PR-TNO/Pt ion-blocking cells.

Table S1. Fractional atomic parameters of TNO with *C2* space group.

atom	*x*	*y*	*z*	occupation	site
Ti1	0	0.25	0	0.04	2*a*
Ti2	0.115091	0	0.000774	0.04	4*c*
Ti3	0.239388	0	0.062323	0.04	4*c*
Ti4	0.368851	0	0.120401	0.04	4*c*
Ti5	0.500509	0	0.167015	0.04	4*c*
Ti6	0.090579	0	0.180923	0.04	4*c*
Ti7	0.218778	0	0.237788	0.04	4*c*
Ti8	0.353744	0	0.310808	0.04	4*c*
Ti9	0.473072	0	0.332711	0.04	4*c*
Ti10	0.077385	0	0.371849	0.04	4*c*
Ti11	0.197283	0	0.414887	0.04	4*c*
Ti12	0.322906	0	0.467499	0.04	4*c*
Ti13	0.448823	0	0.505534	0.04	4*c*
Nb1	0	0.25	0	0.96	2*a*
Nb2	0.115091	0	0.000774	0.96	4*c*

Nb3	0.239388	0	0.062323	0.96	4*c*
Nb4	0.368851	0	0.120401	0.96	4*c*
Nb5	0.500509	0	0.167015	0.96	4*c*
Nb6	0.090579	0	0.180923	0.96	4*c*
Nb7	0.218778	0	0.237788	0.96	4*c*
Nb8	0.353744	0	0.310808	0.96	4*c*
Nb9	0.473072	0	0.332711	0.96	4*c*
Nb10	0.077385	0	0.371849	0.96	4*c*
Nb11	0.197283	0	0.414887	0.96	4*c*
Nb12	0.322906	0	0.467499	0.96	4*c*
Nb13	0.448823	0	0.505534	0.96	4*c*
O1	0.393728	0	0.945934	1	4*c*
O2	0.171185	0	0.004457	1	4*c*
O3	0.314431	0	0.091844	1	4*c*
O4	0.100044	0	0.098983	1	4*c*
O5	0.446448	0	0.139997	1	4*c*
O6	0.225897	0	0.157699	1	4*c*

O7	0.005850	0	0.191246	1	4*c*
O8	0.353599	0	0.225339	1	4*c*
O9	0.149856	0	0.170696	1	4*c*
O10	0.492000	0	0.241404	1	4*c*
O11	0.299403	0	0.335559	1	4*c*
O12	0.071719	0	0.275119	1	4*c*
O13	0.417788	0	0.310014	1	4*c*
O14	0.209981	0	0.333538	1	4*c*
O15	0.335281	0	0.364347	1	4*c*
O16	0.132412	0	0.372285	1	4*c*
O17	0.482196	0	0.430765	1	4*c*
O18	0.254937	0	0.426949	1	4*c*
O19	0.046092	0	0.495464	1	4*c*
O20	0.392769	0	0.506244	1	4*c*
O21	0.179289	0	0.535188	1	4*c*
O22	0.313843	0	0.54858	1	4*c*
O23	0.440186	0	0.592159	1	4*c*

O24	0.961179	0	0.690731	1	4*c*
O25	0.152658	0	0.689249	1	4*c*
O26	0.291363	0	0.725481	1	4*c*
O27	0.415526	0	0.829306	1	4*c*
O28	0.128656	0	0.919867	1	4*c*
O29	0.502828	0	0.910890	1	4*c*
O30	0.253138	0	0.962233	1	4*c*
O31	0.047994	0	0.979671	1	4*c*

Table S2. Fractional atomic parameters of PR-TNO with *A2/m* space group.

atom	*x*	*y*	*z*	occupation	site
Ti1	0.088864	0	0.070469	0.04	4*i*
Ti2	0.095022	0	0.698389	0.04	4*i*
Ti3	0.102465	0	0.884314	0.04	4*i*
Ti4	0.370389	0	0.150956	0.04	4*i*
Ti5	0.360917	0	0.778046	0.04	4*i*
Ti6	0.356660	0	0.955386	0.04	4*i*
Nb1	0.088864	0	0.070469	0.96	4*i*
Nb2	0.095022	0	0.698389	0.96	4*i*
Nb3	0.102465	0	0.884314	0.96	4*i*
Nb4	0.370389	0	0.150956	0.96	4*i*
Nb5	0.360917	0	0.778046	0.96	4*i*
Nb6	0.356660	0	0.955386	0.96	4*i*
O1	0.5	0	0	1	2*d*
O2	0.065352	0	0.180224	1	4*i*
O3	0.069697	0	0.325531	1	4*i*

O4	0.132074	0	0.571586	1	4*i*
O5	0.067405	0	0.770721	1	4*i*
O6	0.108565	0	0.973767	1	4*i*
O7	0.202893	0	0.096665	1	4*i*
O8	0.223185	0	0.726655	1	4*i*
O9	0.196654	0	0.898915	1	4*i*
O10	0.360740	0	0.067983	1	4*i*
O11	0.368366	0	0.257082	1	4*i*
O12	0.349667	0	0.435836	1	4*i*
O13	0.377584	0	0.753276	1	4*i*
O14	0.354039	0	0.846429	1	4*i*
O15	0.497979	0	0.201862	1	4*i*

Table S3. Comparisons of interlayer spacing (*b* value) and charge capacity of PR-TNO at 0.1C with previously-reported M–Nb–O anode compounds having shear ReO_3 crystal structures.

M–Nb–O compound	*b* (Å)	charge capacity (mAh g^{-1})	potential window (V *vs.* Li/Li^+)	reference
**PR-TNO**	**3.8335**	**376**	**3.0–0.8**	**this work**
$ZrNb_{24}O_{62}$	3.8225	280	3.0–0.8	[S1]
$Nb_{12}O_{29}$	3.831	287	3.0–0.8	[S2]
$Nb_{25}O_{62}$	3.82605	289	3.0–0.8	[S2]
$FeNb_{11}O_{29}$	3.82465	266	3.0–0.8	[S3]
$GaNb_{11}O_{29}$	3.80931	255	3.0–0.8	[S4]
$AlNb_{11}O_{29}$	3.81126	267	3.0–0.8	[S5]
$CrNb_{11}O_{29}$	3.8335	286	3.0–0.8	[S6]
$Cr_{0.5}Nb_{24.5}O_{62}$	3.82628	322	3.0–0.8	[S7]
$Mg_2Nb_{34}O_{87}$	3.83071	290	3.0–0.8	[S8]
$Al_{0.5}Nb_{24.5}O_{62}$	3.8228	300	3.0–0.8	[S9]
$Zn_2Nb_{34}O_{87}$	3.8287	284	3.0–0.8	[S10]
$Cu_2Nb_{34}O_{87}$	3.83115	343	3.0–0.8	[S11]
$MoNb_{12}O_{33}$	3.82094	321	3.0–0.8	[S12]

Table S4. Comparison of charge capacity of PR-TNO at 0.1C with previously-reported Ti–Nb–O anode materials.

Ti–Nb–O material	charge capacity (mAh g^{-1})	potential window (V *vs.* Li/Li^{+})	reference
**PR-TNO fibers**	**376**	**3.0–0.8**	**this work**
$TiNb_{24}O_{62}$ bulk	296	3.0–0.8	[S13]
$TiNb_2O_7$ microspheres	243–286	3.0–1.0	[S14,S15]
microsized $Ti_2Nb_{10}O_{29}$ particles	247	2.5–1.0	[S16]
$Ti_{0.95}Mo_{0.05}Nb_2O_7$ bulk	278	3.0–1.0	[S17]
$TiNb_2O_7$ nanofibers	245–280	3.0–1.0	[S18,S19]
$Cr_{0.6}Ti_{0.8}Nb_{10.6}O_{29}$	322	3.0–0.8	[S20]
carbon-coated and surface nitrided $TiNb_2O_7$	268	3.0–1.0	[S21]
$TiNb_6O_{17}$ bulk	284	3.0–1.0	[S22]
$Ti_2Nb_{10}O_{29}$ bulk	280	2.4–1.0	[S23]
nitrided $TiNb_2O_7$ microspheres	265	3.0–1.0	[S18]
porous $Ti_2Nb_{10}O_{29}$ nanospheres	312	2.5–0.8	[S24]
nanoporous $TiNb_2O_7$ nanoparticles	281	3.0–1.0	[S25]

Table S5. Comparisons of apparent Li^+ diffusion coefficient (D_{Li}) of PR-TNO at –20 °C with previously-reported M–Nb–O anode materials at 25 °C.

material	D_{Li} ($cm^2\ s^{-1}$)	test technique	reference
**PR-TNO fibers**	**8.2×10^{-12} (25 °C)**	**GITT**	**this work**
**PR-TNO fibers**	**8.3×10^{-13} (–20 °C)**	**GITT**	**this work**
$W_3Nb_{14}O_{44}$ nanowires	8.02×10^{-13} (25 °C)	CV	[S26]
$Cu_2Nb_{34}O_{87}$ micron-sized particles	3.5×10^{-13} (25 °C)	GITT	[S11]
VNb_9O_{25} nanoribbons	5.17×10^{-15} (25 °C)	EIS	[S27]
$Al_{0.5}Nb_{24.5}O_{62}$ micron-sized particles	2.5×10^{-13} (25 °C)	GITT	[S9]
$MoNb_{12}O_{33}$ micron-sized particles	3.9×10^{-14} (25 °C)	GITT	[S12]
$W_5Nb_{16}O_{55}$ micron-sized particles	1.0×10^{-13} (25 °C)	GITT	[S9]
$GeNb_{18}O_{47}$ nanowires	1.574×10^{-14} (25 °C)	CV	[S28]
$Nb_{18}W_{16}O_{93}$ nanowires	1.312×10^{-14} (25 °C)	EIS	[S29]
$Ru_{0.01}Ti_{0.99}Nb_2O_7$ micron-sized particles	1.66×10^{-15} (25 °C)	EIS	[S30]
$TiNb_2O_7$ nanorods	3.24×10^{-14} (25 °C)	CV	[S31]
$TiCr_{0.5}Nb_{10.5}O_{29}$ nanoparticles	2.07×10^{-14} (25 °C)	CV	[S32]
$Cr_{0.5}Nb_{24.5}O_{62}$ micron-sized particles	4.57×10^{-14} (25 °C)	EIS	[S7]
Nb_2O_5 nanorods	3.66×10^{-17} (25 °C)	CV	[S33]

Equation S1. According to the Tauc plot method, the following equation is used to calculate the band gap energy:[S35]

$$(\alpha h\nu)^n = K(h\nu - E_g)$$

where α is the absorption coefficient, K is the energy independence coefficient, $h\nu$ is the incident photon energy, and E_g is the band gap energy. The band gap energy is decreased from 3.20 eV to 2.85 eV when TNO is partially reduced to PR-TNO.

Equation S2: [S10,S35]

$$D = \frac{4}{\pi\tau}\left(\frac{n_\mathrm{m}V_\mathrm{m}}{S}\right)^2\left(\frac{\Delta E_\mathrm{s}}{\Delta E_\tau}\right)^2 \qquad \left(\tau \ll \frac{L^2}{D^2}\right)$$

where D is the apparent diffusion coefficient, τ is the relaxation time, n_m is the mole number of the electrode material, V_m is the molar volume of the electrode material, S is the contact area between the electrode and the electrolyte, L is the electrode thickness, ΔE_s is the potential change caused by the pulse, and ΔE_τ is the potential change of the constant current discharge or charge.

References

[S1]C. Yang, Y. Zhang, F. Lv, C. Lin, Y. Liu, K. Wang, J. Feng, X. Wang, Y. Chen, J. Li, S. Guo, Porous $ZrNb_{24}O_{62}$ nanowires with pseudocapacitive behavior achieve high-performance lithium-ion storage, J. Mater. Chem. A 5 (2013) 22297–22304.

[S2]R. Li, Y. Qin, X. Liu, L. Yang, C. Lin, R. Xia, S. Lin, Y. Chen, J. Li, Conductive $Nb_{25}O_{62}$ and $Nb_{12}O_{29}$ anode materials for use in high-performance lithium-ion storage, Electrochim. Acta 266 (2018) 202–211.

[S3]X. Lou, Z. Xu, Z. Luo, C. Lin, C. Yang, H. Zhao, P. Zheng, J. Li, N. Wang, Y. Chen, H. Wu, Exploration of $Cr_{0.2}Fe_{0.8}Nb_{11}O_{29}$ as an advanced anode material for lithium-ion batteries of electric vehicles, Electrochim. Acta 245 (2017) 482–488.

[S4]X. Lou, Q. Fu, J. Xu, X. Liu, C. Lin, J. Han, Y. Luo, Y. Chen, X. Fan, J. Li, $GaNb_{11}O_{29}$ nanowebs as high-performance anode materials for lithium-ion batteries, ACS Appl. Nano Mater. 1 (2017) 183–190.

[S5]X. Lou, R. Li, X. Zhu, L. Luo, Y. Chen, C. Lin, H. Li, X.S. Zhao, New anode material for lithium-ion batteries: aluminum niobate ($AlNb_{11}O_{29}$), ACS Appl. Mater. Interfaces 11 (2019) 6089–6096.

[S6]Q. Fu, X. Liu, J. Hou, Y. Pu, C. Lin, L. Yang, X. Zhu, L. Hu, S. Lin, L. Luo, Y. Chen, Highly conductive $CrNb_{11}O_{29}$ nanorods for use in high-energy, safe, fast-charging and stable lithium-ion batteries, J. Power Sources 397 (2018) 231–239.

[S7]C. Yang, S. Yu, C. Lin, F. Lv, S. Wu, Y. Yang, W. Wang, Z.Z. Zhu, J. Li, N. Wang, S. Guo, $Cr_{0.5}Nb_{24.5}O_{62}$ nanowires with high electronic conductivity for high-rate and long-life lithium-ion storage, ACS Nano 11 (2017) 4217–4224.

[S8]X. Zhu, Q. Fu, L. Tang, C. Lin, J. Xu, G. Liang, R. Li, L. Luo, Y. Chen, $Mg_2Nb_{34}O_{87}$ porous microspheres for use in high-energy, safe, fast-charging, and stable lithium-ion batteries, ACS Appl. Mater. Interfaces 10 (2018) 23711–23720.

[S9]Q. Fu, R. Li, X. Zhu, G. Liang, L. Luo, Y. Chen, C. Lin, X.S. Zhao, Design, synthesis and lithium-ion storage capability of $Al_{0.5}Nb_{24.5}O_{62}$, J. Mater. Chem. A 7 (2019) 19862–19871.

[S10]X. Zhu, H. Cao, R. Li, Q. Fu, G. Liang, Y. Chen, L. Luo, C. Lin, X.S. Zhao, Zinc niobate materials: crystal structures, energy-storage capabilities and working mechanisms, J. Mater. Chem. A 7 (2019) 25537–25547.

[S11]L. Yang, X. Zhu, X. Li, X. Zhao, K. Pei, W. You, X. Li, Y. Chen, C. Lin, R. Che, Conductive copper niobate: superior Li^+-storage capability and novel Li^+-transport

mechanism, Adv. Energy Mater. 9 (2019) 1902174.
[S12]X. Zhu, J. Xu, Y. Luo, Q. Fu, G. Liang, L. Luo, Y. Chen, C. Lin, X.S. Zhao, $MoNb_{12}O_{33}$ as a new anode material for high-capacity, safe, rapid and durable Li^+ storage: structural characteristics, electrochemical properties and working mechanisms, J. Mater. Chem. A 7 (2019) 6522–6532.
[S13]C. Yang, S. Deng, C. Lin, S. Lin, Y. Chen, J. Li, H. Wu, Porous $TiNb_{24}O_{62}$ microspheres as high-performance anode materials for lithium-ion batteries of electric vehicles, Nanoscale 8 (2016) 18792–18799.
[S14]H. Park, H.B. Wu, T. Song, X.W. Lou, U. Paik, Porosity-controlled $TiNb_2O_7$ microspheres with partial nitridation as a practical negative electrode for high-power lithium-ion batteries, Adv. Energy Mater. 5 (2015) 1401945.
[S15]H. Li, L. Shen, G. Pang, S. Fang, H. Luo, K. Yang, X. Zhang, $TiNb_2O_7$ nanoparticles assembled into hierarchical microspheres as high-rate capability and long-cycle-life anode materials for lithium ion batteries, Nanoscale 7 (2015) 619–624.
[S16]X. Wu, J. Miao, W. Han, Y.S. Hu, D. Chen, J.S. Lee, J. Kim, L. Chen, Investigation on $Ti_2Nb_{10}O_{29}$ anode material for lithium-ion batteries, Electrochem. Commun. 25 (2012) 39–42.
[S17]H. Song, Y.T. Kim, A Mo-doped $TiNb_2O_7$ anode for lithium-ion batteries with high rate capability due to charge redistribution, Chem. Commun. 51 (2015) 9849–9852.
[S18]H. Park, T. Song, U. Paik, Porous $TiNb_2O_7$ nanofibers decorated with conductive $Ti_{1-x}Nb_xN$ bumps as a high power anode material for Li-ion batteries, J. Mater. Chem. A 3 (2015) 8590–8596.
[S19]X. Wang, Guozhen Shen, Intercalation pseudo-capacitive $TiNb_2O_7$@carbon electrode for high-performance lithium ion hybrid electrochemical supercapacitors with ultrahigh energy density, Nano Energy 15 (2015) 2211–2855.
[S20]C. Yang, S. Yu, Y. Ma, C. Lin, Z. Xu, H. Zhao, S. Wu, P. Zheng, Z.Z. Zhu, J. Li, N. Wang, Cr^{3+} and Nb^{5+} co-doped $Ti_2Nb_{10}O_{29}$ materials for high-performance lithium-ion storage, J. Power Sources 360 (2017) 470–479.
[S21]J. Gao, X. Cheng, S. Lou, X. Wu, F. Ding, P. Zuo, Y. Ma, C. Du, Y. Gao, G. Yin, Surface nitrided and carbon coated $TiNb_2O_7$ anode material with excellent performance for lithium-ion batteries, J. Alloys Compd. 835 (2020) 155241.
[S22]Y.S. Lee, K.S. Ryu, Study of the lithium diffusion properties and high rate performance of $TiNb_6O_{17}$ as an anode in lithium secondary battery, Sci. Rep. 7 (2017) 16617.
[S23]Q. Cheng, J. Liang, Y. Zhu, L. Si, C. Guo, Y. Qian, Bulk $Ti_2Nb_{10}O_{29}$ as long-life and high-power Li-ion battery anodes, J. Mater. Chem. A 2 (2014) 17258–17262.
[S24]X. Xia, S. Deng, S. Feng, J. Wu, J. Tu, Hierarchical porous $Ti_2Nb_{10}O_{29}$ nanospheres as superior anode materials for lithium ion storage, J. Mater. Chem. A 5 (2017) 21134–21139.
[S25]B. Guo, X. Yu, X.G. Sun, M. Chi, Z.A. Qiao, J. Liu, Y.S. Hu, X.Q. Yang, J.B. Goodenough, S. Dai, A long-life lithium-ion battery with a highly porous $TiNb_2O_7$ anode for large-scale electrical energy storage, Energy Environ. Sci. 7 (2014) 2220–2226.
[S26]L. Yan, J. Shu, C. Li, X. Cheng, H. Zhu, H. Yu, C. Zhang, Y. Zheng, Y. Xie, Z. Guo, $W_3Nb_{14}O_{44}$ nanowires: Ultrastable lithium storage anode materials for advanced rechargeable batteries, Energy Storge Mater. 16 (2019) 535–544.
[S27]S. Qian, H. Yu, L. Yan, H. Zhu, X. Cheng, Y. Xie, N. Long, M. Shui, J. Shu, High-rate long-life pored nanoribbon VNb_9O_{25} built by interconnected ultrafine nanoparticles as anode for lithium-ion batteries, ACS Appl. Mater. Interfaces 9 (2017) 30608–30616.
[S28]F. Ran, X. Cheng, H. Yu, R. Zheng, T. Liu, X. Li, N. Ren, M. Shui, J. Shu, Nano-structured $GeNb_{18}O_{47}$ as novel anode host with superior lithium storage performance, Electrochim. Acta 282 (2018) 634–641.

[S29]W. Ye, H. Yu, X. Cheng, H. Zhu, R. Zheng, T. Liu, N. Long, M. Shui, J. Shu, Highly efficient lithium container based on non-Wadsley-Roth structure $Nb_{18}W_{16}O_{93}$ nanowires for electrochemical energy storage, Electrochim. Acta 292 (2018) 331−338.
[S30]C. Lin, S. Yu, S. Wu, S. Lin, Z.Z. Zhu, J. Li, L. Lu, $Ru_{0.01}Ti_{0.99}Nb_2O_7$ as an intercalation-type anode material with a large capacity and high rate performance for lithium-ion batteries, J. Mater. Chem. A 3 (2015) 8627−8635.
[S31]L. Hu, C. Lin, C. Wang, C. Yang, J. Li, Y. Chen, S. Lin, $TiNb_2O_7$ nanorods as a novel anode material for secondary lithium-ion batteries, Funct. Mater. Lett. 9 (2017) 1642004.
[S32]L. Hu, R. Lu, L. Tang, R. Xia, C. Lin, Z. Luo, Y. Chen, J. Li, $TiCr_{0.5}Nb_{10.5}O_{29}$/CNTs nanocomposite as an advanced anode material for high-performance Li^+-ion storage, J.Alloys Compd. 732 (2018) 116−123.
[S33]C. Shi, K. Xiang, Y. Zhu, W. Zhou, X. Chen, H. Chen, Box-implanted Nb_2O_5 nanorods as superior anode materials in lithium ion batteries, Ceram. Int. 43 (2017) 12388−12395.
[S34]J. Tauc, R. Grigorovic, A. Vancu, Optical properties and electronic structure of amorphous germanium, Phys. Status Solidi. 15 (1966) 627−637.
[S35]A.J. Bard, L.R. Faulkner, Electrochemical Methods: Fundamentals and Applications, second ed., Wiley, New York, 2001.
